# Renal vein thrombosis associated with oral contraception and smoking: a case report from Japan, with literature review

**DOI:** 10.1007/s13730-013-0095-9

**Published:** 2013-09-04

**Authors:** Yosuke Sasaki, Akira Shimabukuro, Takuya Isegawa, Yuiichi Tamori, Taro Koshiishi, Hiroyasu Yonaha

**Affiliations:** 1 Department of Internal Medicine, Okinawa Yaeyama Hospital, 732 Okawa, Ishigaki, Okinawa 907-0022 Japan; 2 Department of Obstetrics and Gynecology, Okinawa Yaeyama Hospital, 732 Okawa, Ishigaki, Okinawa 907-0022 Japan; 3 Department of General Medicine, Okinawa Yaeyama Hospital, 732 Okawa, Ishigaki, Okinawa 907-0022 Japan

**Keywords:** Renal vein thrombosis, Oral contraceptives, Smoking, Asian ethnicity, Flank pain

## Abstract

Renal vein thrombosis, one of the common thrombotic complications of nephrotic syndrome or renal cell carcinoma, is reportedly a rare complication of hormonal contraception. Solitary renal vein thrombosis in the Japanese population is thought to be very rare because the incidence of venous thromboembolism is comparatively very low in Asian populations. We report a 38-year-old Japanese female with left renal vein thrombosis associated with oral contraception and concurrent smoking as the first Japanese case of solitary renal vein thrombosis associated with oral contraceptives, with a review of the literature. Seven cases were previously reported. The results revealed that all patients complained of acute onset of pain around the involved kidney without urinary symptoms or fever, and were effectively treated with anticoagulants. Other remarkable facts include that nausea and vomiting were frequently seen, and that the renal outcome was benign, despite various initial urine abnormalities. This report may alert clinicians to the importance of these risk factors as an etiology of renal vein thrombosis even in Asian populations. Clinicians should regard renal vein thrombosis as one of the differential diagnoses for acute flank pain in patients using oral contraceptives. A detailed history taking that reveals oral contraception, smoking, and other thrombophilic predispositions as well as timely computed tomographic scans would be the keys to diagnosis. Smoking cessation should be strongly recommended to oral contraceptive users, especially women over 35 years of age, regardless of dosage.

## Introduction

Renal vein thrombosis (RVT) is currently considered to be a rare complication of hormonal contraception [1]. The incidence of any type of venous thromboembolism (VTE) is very low in Asians compared to that in other ethnicities [2]. Here, we report the first Japanese case of solitary RVT associated with oral contraceptives (OC) and concurrent smoking, and review the literature of previously reported cases of RVT associated with OC.

## Case report

A 38-year-old Japanese female smoker with a previous history of OC use visited the emergency department with a chief complaint of a sudden onset of severe left flank pain. Her present history started with exacerbation of dysmenorrhea, which induced her to take loxoprofen sodium 60 mg tid for the previous 10 days. Five days prior to admission, she also restarted taking an OC, drospirenone/ethinyl estradiol (YAZ), for dysmenorrhea due to adenomyosis uteri. On the morning of admission, she was awoken by severe left flank pain, a continuous aching pain, roughly 6/10 on the conventional pain scale. She also had nausea that started immediately after the onset of the chief compliant. She had noticed dyspnea on effort during the previous 3 months but denied other symptoms such as chest pain, back pain, constipation, diarrhea, tarry stools, or hematochezia. A review of her symptoms revealed anorexia accompanied by a 5-kg loss of body weight over the previous 3 months.

Two years prior to her emergency department visit, the patient was diagnosed with adenomyosis uteri, myoma uteri, and iron deficiency anemia due to hypermenorrhea at that time. One year prior, she was prescribed YAZ, a low-dose OC, for dysmenorrhea and hypermenorrhea due to adenomyosis, but discontinued this medication 4 months before admission due to diminished symptoms, until 5 days prior to her hospital visit. She had tried to take iron tablets 2 years prior, but failed due to nausea. She had one induced abortion at the age of 18. Her recent menstruations were regular, the last starting 2 weeks prior and finishing 1 week prior to admission, 2 days before she started taking OC again. She was currently a smoker, and had smoked ten cigarettes per day for 10 years. She denied smoking more than 15 cigarettes per day. She had divorced several years prior and currently lived with her parents. She managed a bar and consumed a large amount of alcohol every night at the bar. She had no family history of thromboembolic disorders or malignancies.

The patient’s height was 160 cm, body weight 47.4 kg, and body mass index 18.5 kg/m^2^. She was alert and cooperative but annoyed by her pain and appeared ill. Her blood pressure was 126/70 mmHg, pulse regular at 86 beats per min, respiration 24 breaths per min, body temperature 37.2 °C, and blood oxygen saturation level on room air was 100 %. Her face and conjunctivae appeared pale, but not icteric, and her oral cavity showed no aphthae or petechiae. There was no lymphadenopathy, bruits, or venous hum, her heart sounds were regular, without murmur or gallops, and lung sounds indicated a bilaterally clear condition. Abdominal findings showed tenderness at the left flank area with obvious rebound and muscular guarding. Light percussion at the left costovertebral angle provoked knocking pain. No edema, petechiae, or peripheral signs were observed.

**Fig. 1:**
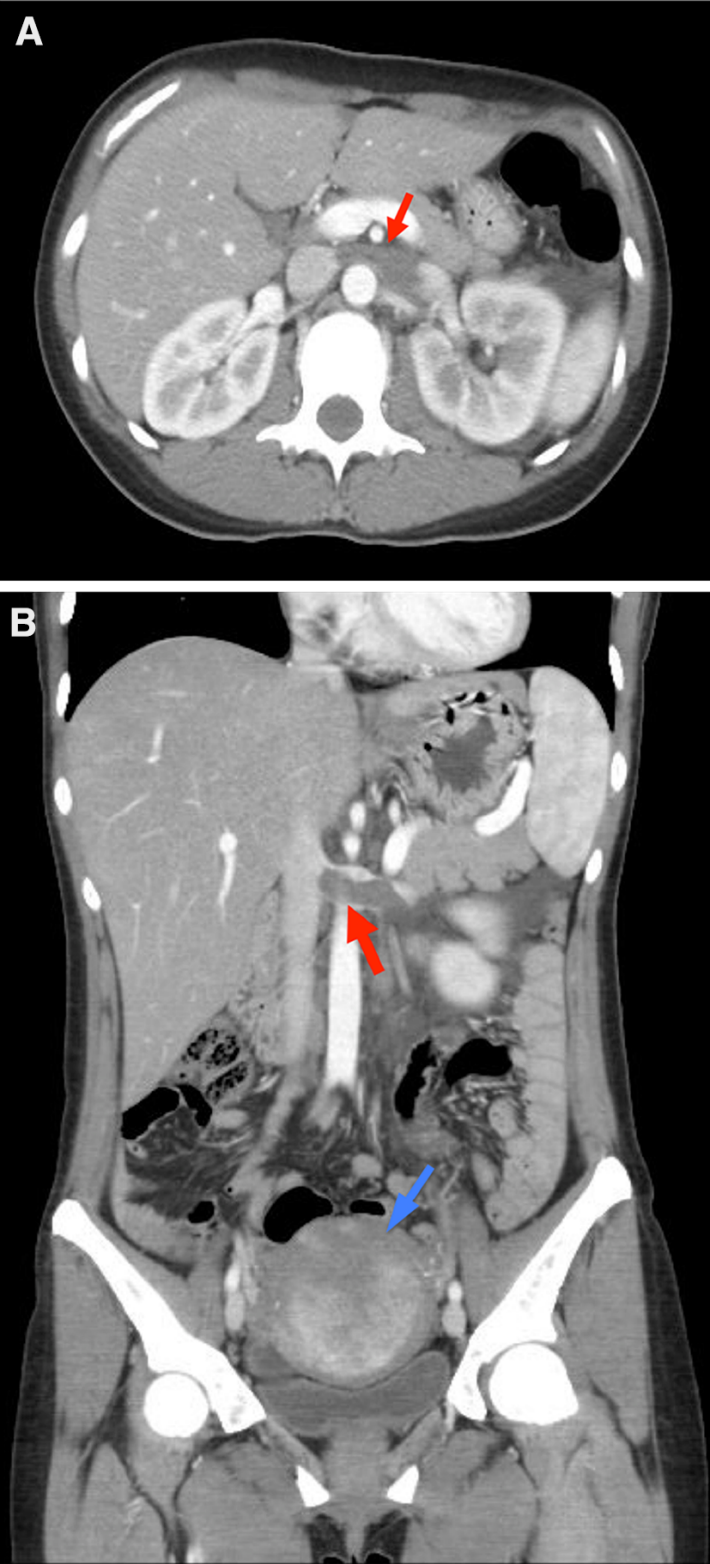
**a** An axial image of abodominal CT scan with contrast material. A large thrombus occluded the left renal vein (*red arrow*). **b** A coronal image of abdominal CT scan with contrast material. A large thrombus occluded the left renal vein (*red arrow*). The uterus was enlarged due to adenomyosis uteri and myoma uteri (*blue arrow*)

Laboratory data (Table 1) was remarkable for severe anemia. Other routine data, including renal function, liver function, and electrolytes, were normal. Urinalysis revealed proteinuria (1.4 g/gCr) and microhematuria. Urine sediment showed 1–4 neutrophils, 10–19 red cells, and 1–4 squamous epitheliums per high-powered field (HPF), without bacteria or casts. Left renal vein thrombosis was seen on computed tomographic (CT) scan with contrast material and echogram of the abdomen (Fig. 1a, b). No other thrombi, masses, or apparent compression of the renal vein suggesting nutcracker syndrome were seen.

**Table 1:** Laboratory data

Parameters	Day of admission	Day of discharge (18 days later)
White blood cell (/mm^3^)	5,880	5,560
Hemoglobin (g/dL)	5.8	12.5
Hematocrit (%)	22.5	38.8
Platelet (×10^4^/mm^3^)	30.6	30.3
Sodium (mEq/L)	140	137
Potassium (mEq/L)	3.5	4.2
Chloride (mEq/L)	103	106
BUN (mg/dL)	8.8	10.6
Creatinine (mg/dL)	0.5	0.4
AST (IU/L)	20	45
ALT (IU/L)	25	83
LDH (IU/l)	179	184
Albumin (g/dL)	3.8	N/A
PT-INR	1.03	3
aPTT/aPTT-ST (s)	26.8/30.0	N/A
Fibrinogen (mg/dL)	229	N/A
FDP (μg/ml)	5	N/A
vWF antigen (%)	126	N/A
Factor V coagulation activity (%)	92.1	N/A
Protein C activity (%)	96	N/A
Protein C antigen (%)	84	N/A
Protein S (%)	69.3	N/A
Protein S antigen (%)	65	N/A
Anti-nuclear antibody (EIA)	7.9	N/A
Anti-cardiolipin IgG (U/mL)	<1	N/A
Anti-CL-β2GP1 complex antibody (U/mL)	<1.3	N/A
LAC/DRVVT	1	N/A
Anti-ds-DNA antibody IgG (U/mL)	<0.5	N/A
Anti-Sm antibody (U/mL)	<0.5	N/A
Anti SS-A/Ro antibody (U/mL)	<0.5	N/A
Anti SS-B/La antibody (U/mL)	<0.5	N/A
Urine		
Red blood cell (/HPF)	10–19	<1
White blood cell (/HPF)	1–4	<1
Protein (g/gCr)	1.4	<0.3

*ALT* alanine aminotransferase, *Anti-CL-β2GP1 complex antibody* anti-cardiolipin β2 glycoprotein-1 complex antibody, *AST* aspartate aminotransferase, *aPTT* activated partial thromboplastin time, *BUN* blood urea nitrogen, *EIA* enzyme immune assay, *FDP* fibrin degradation product, *LAC/DRVVT* lupus anticoagulant/dilute Russell’s viper venom time ratio, *LDH* lactate dehydrogenase, *PT-INR* prothrombin time-international normalized ratio, *vWF* von Willebrand factor

Drospirenone/ethinyl estradiol was discontinued immediately and acetaminophen was administered instead of loxoprofen sodium. Anticoagulation therapy with continuous intravenous heparin and subsequent oral warfarin was initiated immediately after laboratory specimens were submitted to evaluate underlying thrombophilic predispositions. Concentrated red cells were transfused for symptomatic anemia and a tolerable dosage of iron tablets was started.

Due to the patient’s anorexia and body weight loss, we performed upper and lower gastrointestinal endoscopy, but there were no findings suggesting ulcers, inflammation, active bleeding, or malignancies. Gynecological examination and transvaginal echogram found no malignancies. Blood examinations submitted before these procedures revealed no underlying thrombophilia such as connective tissue diseases, vasculitis syndromes, anti-phospholipid syndrome, factor V Leiden deficiency, anti-thrombin deficiency, protein S deficiency, protein C deficiency, or disseminated intravascular coagulation (Table 1). Repeated urinalysis and normal serum albumin excluded nephrotic syndrome. Abdominal ultrasound, performed after admission, confirmed the thrombus at the left renal vein and excluded other thromboembolism or findings of nutcracker syndrome.

The patient’s flank pain and anorexia improved soon after treatment was begun. A CT scan performed 2 weeks after admission showed remarkable volume reduction of the thrombus and the patient was discharged, with instructions to continue warfarin and iron supplement administration. Although transient breakthrough bleeding and macrohematuria continued for a few days after admission, no complications such as pulmonary embolism or persistent bleeding occurred during treatment. The patient’s urine findings were normalized, with hemoglobin at 12.5 g/dL and PT-INR extended to 3.0 at the time of discharge, 18 days after admission (Table 1). Elevated transaminases at the day of discharge were spontaneously normalized at the outpatient clinic without any intervention. The patient stopped smoking, took no OC after admission, and was in good condition during our continuous observation at the outpatient clinic.

## Literature review

The PubMed database was searched for related literature, using ‘renal vein thrombosis’ and ‘contraceptives’ as search parameters. Ichushi-Web was also searched for literature written in Japanese, using ‘renal vein thrombosis’ (jinjoumyakukessen) as the search parameter.

The searches revealed seven prior cases, including one Japanese case complicated with portal and mesenteric vein thrombosis (3–9). Table 2 provides detailed information of these cases and includes our case. Age at onset ranged from 15 to 47 years (median, 24.5 years). The right renal vein was involved in 3/8 (37.5 %) cases and the left renal vein was involved in 5/8 (62.5 %) cases. No case was complicated with any other thromboembolism except for case 7, which was complicated with a massive portal and mesenteric thrombosis. Patients 1 and 3 had received norethindrone/ethinyl estradiol (Ortho-Novum) and our patient had taken YAZ; we could not obtain information about medications used in other patients. Periods of OC use were reported in five cases, with a very wide temporal distribution (5 days to 10 years). Our case was remarkable for the very short duration of OC use compared to that in the other cases. Patient 2 had one pregnancy and one abortion; patient 6 had two pregnancies and one elective abortion; our case had one induced abortion. Patient 4 did not smoke; our patient smoked 10 cigarettes per day for 20 years; no information about smoking was obtained in the other patients. Flank pain was present in all cases. Nausea/vomiting was seen in 5/8 cases (62.5 %). No patients complained of urinary symptoms or had fever higher than 38.0 °C. Only patient 1, who developed refractory hypertension and acute heart failure, had remarkable hypertension on admission; blood pressures were within normal ranges in the other patients. Proteinuria and hematuria were seen in most cases, with various severities. Renal function was transiently impaired in some cases but eventually recovered in all cases; renal outcome was good in all cases.

**Table 2:** Profiles of cases with renal thrombosis associated with oral contraceptives

Patient	Age	Country	Ethnicity	Involved side	Duration of contraceptive use	Past medical history	Symptoms	Complications	Treatment	Years	Author
1	24	USA	Caucasian	Left	4 years	None	Flank pain	Hypertension CHF, ARF	N/R	1975	Slick et al. [3]
2	15	USA	N/R	Left	N/R	None	Flank pain, N/V	None	Anticoagulation	1983	Goldman et al. [4]
3	25	USA	N/R	Right	5 years	IDDM	Flank pain, N/V	None	Systemic UK injection	1986	Barre et al. [5]
4	20	Germany	Caucasian	Left	8 months	Renal stone	Flank pain, N/V	Dehydration	Local UK injection	1989	Boehler et al. [6]
5	21	Canada	N/R	Right	6 months	IDA Hypermenorrhea	Flank pain, N/V	None	Heparin and subsequent WF	2001	Chan et al. [7]
6	35	USA	N/R	Right	10 years	None	Flank pain	None	Heparin and subsequent WF	2010	Ajmera et al. [8]
7	47	Japan	Asian	Left	N/R	None	Flank pain	Portal vein thrombosis	Anticoagulation	2013	Iwasaki et al. [9]
8	38	Japan	Asian	Left	5 days (previously, 1 year)	IDA Adenomyosis uteri Myoma uteri	Flank pain, N/V	Anemia Dehydration	Heparin and subsequent WF	2013	Sasaki et al.^a^

*ARF* acute renal failure, *CHF* congestive heart failure, *IDA* iron deficiency anemia, *IDDM* insulin-dependent diabetes mellitus, *N/R* not recorded, *N/V* nausea and/or vomiting, *UK* urokinase, *WF* warfarin

^a^Present case

## Discussion

RVT usually presents as an insidious, chronic disease in patients with nephrotic syndrome or renal cell carcinoma. Acute RVT is a rare condition and usually occurs after blunt abdominal trauma or renal transplantation (4). A large cohort study performed in Denmark revealed that RVT including caval thrombosis occurs in only 0.8 % of all VTE cases associated with hormonal contraception [1]. Although ethnicity data is not available in this study, it nevertheless suggests that renal vein thrombosis is a rare complication even in European countries, where Caucasians are the majority.

There is strong evidence that Asians/Pacific Islanders have a significant three- to five-fold lower incidence of symptomatic idiopathic and secondary VTE. Furthermore, this racial group has significantly lower incidence of cancer-associated VTE, but the etiology of this epidemiological difference is still unclear [10]. Specific genetic conditions, particularly the presence of factor V Leiden, or the prothrombin gene mutation (G20210A), are known to be associated with a three- to four-fold higher risk for developing VTE. Because these two genetic traits are much more prevalent in Caucasian populations than in Asian populations, this fact may explain the higher prevalence of VTE in Caucasian populations [2]. Interestingly, studies from Asia suggest that the incidence of asymptomatic VTE is comparable to that in North America, suggesting that a much lower percentage of Asians transition from asymptomatic to symptomatic VTE. These studies revealed that Asian patients do frequently form small calf clots, but they have a lower incidence of symptomatic VTE. One hypothesis for this observation is that Asians have a more active or efficient fibrinolytic system [2, 10].

Although evidence concerning the relationship between smoking and VTE is conflicting, some studies have detected increased relative risks of VTE in smokers, ranging up to 3.3 times greater [11]. However, epidemiologic and case–control studies carried out during the past 40 years have produced inconsistent evidence that concurrent smoking and OC use is associated with an increase in cardiovascular diseases such as myocardial infarction [12]. Given these facts, the World Health Organization (WHO) has suggested that OC should not be taken by women over 35 years of age who smoke more than 15 cigarettes per day, but that OC can be considered in women older than 35 years who smoke fewer than 15 cigarettes per day, since the risk of pregnancy in this age group is greater than risks associated with OC use. Japanese guidelines follow the WHO recommendation [13], but the American College of Obstetricians and Gynecologists recommends that physicians prescribe combination OC with caution, if at all, in women older than 35 years who smoke, regardless of the number of cigarettes per day, because patients often under-report the number [14]. Furthermore, it may be advisable for clinicians to more carefully consider OC use other than for contraception, such as for dysmenorrhea or hypermenorrhea, because evidence concerning OC efficacies for these conditions is still inconsistent, although it worked well in our case [15].

Thus, despite our careful evaluation concerning possible predispositions, our patient had no other risk factors for RVT other than OC use and concurrent smoking of less than 15 cigarettes per day. Given that our review of the literature revealed only six cases of solitary RVT, and that the only Japanese case reported was a conference report of RVT associated with massive portal vein and mesenteric vein thrombosis, our case presented here is the first Japanese case of solitary RVT associated with OC and concurrent smoking.

Given her lean body shape and dehydrated status due to persistent anorexia, we believe that the hemodynamic congestion at the left renal vein due to extra-luminal compression caused by the adjacent superior mesenteric artery and abdominal aorta may have contributed to form the thrombus at the left renal vein without any other thromboses. Although the compression of the left renal vein was not apparent on imaging studies, we think that the hemodynamic congestion might have contributed as a composition of complicated predispositions including stronger factors such as OC use and smoking. The facts that RVT tended to occur at the left renal vein in past cases and that the onset of our case was during sleep in the supine position support this hypothesis.

The association between the duration of OC use and the development of thromboembolic complications is an important point of discussion in this case report. OC use is generally regarded as a thrombophilic factor as it elicits the intrinsic thrombophilic predisposition of the user and reportedly increases the risk of VTE within 3 months after initiation [16, 17]. We regarded the relevant duration of OC use as 5 days because the patient had discontinued previous OC use 4 months prior to the episode and the risk of thromboembolism associated with OC is generally thought to decrease to the same level as that of a non-OC user within 3 months after discontinuation [17]. However, considering the much shorter duration of OC use in the present case compared to previously reported cases, we suspect that there are certain effects of past OC use (which started 1 year ago and discontinued 4 months ago) involved in this episode as well as coexisting factors such as smoking, dehydrated status, and lean body shape that can predispose individuals to left renal vein compression due to adjacent vessels, as discussed above.

According to the review of the cases, the typical clinical course of RVT associated with OC use can be concisely described as follows: a fertile-aged woman, with no histories of preceding abdominal trauma or renal transplantation, visits a hospital with chief complaint of acute severe flank pain, frequently accompanied by nausea and vomiting, without urinary symptoms or fever. Urinary findings are various and non-specific for RVT. Blood examination is not diagnostic for RVT but necessary to exclude other fatal diseases. Anticoagulants seem effective with good renal outcome. A detailed history taking that reveals OC use, smoking, and other thrombophilic predispositions would be the key to diagnosis. Considering that the clinical manifestation of RVT seems indistinguishable from other fatal causes of ‘acute abdomen’, timely CT scan is useful in the diagnosis and exclusion of other causes or complications. Careful radiographic evaluation of the veins as well as arteries and other organs is required.

Even though VTE associated with OC or smoking is a rare complication in Asian populations, our experience may alert clinicians to the importance of these risk factors as an etiology of RVT. Solitary renal vein thrombosis should be included in the differential diagnosis of acute abdominal pain, flank pain in particular, and in patients using oral contraceptives. Smoking cessation is therefore strongly recommendable to OC users, especially women over 35, regardless of the dosage. Further study concerning VTE including RVT associated with OC use in Asian populations is warranted.
